# Bedside rationing and moral distress in nephrologists in sub- Saharan Africa

**DOI:** 10.1186/s12882-022-02827-2

**Published:** 2022-05-25

**Authors:** Gloria Ashuntantang, Ingrid Miljeteig, Valerie A. Luyckx

**Affiliations:** 1 Yaoundé General Hospital Faculty of Medicine & Biomedical Sciences, University of Yaoundé I, Yaoundé, Cameroon; 2 Faculty of Health Sciences, The University of Bamenda, Bamenda, Cameroon; 3 Bergen Centre for Ethics and Priority Setting, Department of Global Public Health and Primary Care, University of Bergen, Bergen, Norway; 4 Department of Research and Development, Helse Bergen Health Trust, Bergen, Norway; 5 Department of Paediatrics and Child Health, University of Cape Town, Cape Town, South Africa; 6 Renal Division, Brigham and Women’s Hospital, Harvard medical School, Boston, MA USA; 7 Department of Nephrology, University Children’s Hospital, Zurich, Switzerland

**Keywords:** Moral distress, Ethics, Dialysis, Sub-Saharan Africa, Rationing, Nephrology, Priority setting, Physicians, Financial risk protection, Catastrophic health expenditure (3–10)

## Abstract

**Background:**

Kidney diseases constitute an important proportion of the non-communicable disease (NCD) burden in Sub-Saharan Africa (SSA), though prevention, diagnosis and treatment of kidney diseases are less prioritized in public health budgets than other high-burden NCDs. Dialysis is not considered cost-effective, and for those patients accessing the limited service available, high out-of-pocket expenses are common and few continue care over time. This study assessed challenges faced by nephrologists in SSA who manage patients needing dialysis. The specific focus was to investigate if and how physicians respond to bedside rationing situations.

**Methods:**

A survey was conducted among a randomly selected group of nephrologists from SSA. The questionnaire was based on a previously validated survey instrument. A descriptive and narrative approach was used for analysis.

**Results:**

Among 40 respondents, the majority saw patients weekly with acute kidney injury (AKI) or end-stage kidney failure (ESKF) in need of dialysis whom they could not dialyze. When dialysis was provided, clinical compromises were common, and 66% of nephrologists reported lack of basic diagnostics and medication and > 80% reported high out-of-pocket expenses for patients. Several patient-, disease- and institutional factors influenced who got access to dialysis. Patients’ financial constraints and poor chances of survival limited the likelihood of receiving dialysis (reported by 79 and 78% of nephrologists respectively), while a patient’s being the family bread-winner increased the likelihood (reported by 56%). Patient and institutional constraints resulted in most nephrologists (88%) frequently having to make difficult choices, sometimes having to choose between patients. Few reported existence of priority setting guidelines. Most nephrologists (74%) always, often or sometimes felt burdened by ethical dilemmas and worried about patients out of hospital hours. As a consequence, almost 46% of nephrologists reported frequently regretting their choice of profession and 26% had considered leaving the country.

**Conclusion:**

Nephrologists in SSA face harsh priority setting at the bedside without available guidance. The moral distress is high. While publicly funded dialysis treatment might not be prioritized in essential health care packages on the path to universal health coverage, the suffering of the patients, families and the providers must be acknowledged and addressed to increase fairness in these decisions.

**Supplementary Information:**

The online version contains supplementary material available at 10.1186/s12882-022-02827-2.

## Introduction

The COVID-19 pandemic has highlighted the ethical dilemmas faced daily by clinicians when trying to manage high volumes of severely ill patients under conditions of resource constraints. These experiences in high income settings have resulted in front page news [1], rapid development of surge contingencies [2], and in the global acceptance of triage guidelines for access to intensive care treatment [3], which under other circumstances would be highly contested. Qualitative studies have highlighted the discomfort of physicians at the bedside, in countries with health systems generally characterized by plenty, who had to make compromises in care, and who recognized that in trying to stretch resources “everyone gets a little bit of bad care” [4]. These dilemmas however are not new. They are very familiar to nurses and to physicians practicing daily under resource limitations [5–8]. In most lower-income settings health care providers must deal with high disease burdens, small health budgets, limited public services and shortages of trained colleagues, as well as poor populations and geographical and logistical barriers on a daily basis [9, 10].

Many countries in sub-Saharan Africa (SSA) have experienced a rapid increase in non-communicable diseases [NCDs] over the past decade [11]. Kidney disease is an important contributor to this burden [12], with chronic kidney disease (CKD) being the 5th most common cause of NCD deaths in the region, after stroke, cardiovascular disease, cirrhosis and diabetes (https://vizhub.healthdata.org/gbd-compare/). Managing patients in need of chronic care poses a challenge to already strained health systems still dealing with high levels of infections and maternal-child conditions [13]. Although kidney diseases constitute an important proportion of the NCD disease burden in SSA, prevention, diagnosis and treatment are not highly prioritized in public health budgets [14, 15].

Two systematic reviews have highlighted the challenges in access to care for patients with acute or end-stage kidney failure who require dialysis in SSA [16, 17]. Although dialysis was indicated in a high proportion of adults and children with acute kidney injury (AKI), only around 1 in 3 gained access to dialysis in most countries [17]. Mortality among those not dialyzed when needed with this largely reversible condition was high (73% in children, 86% in adults). Among patients with end-stage kidney failure (ESKF), pooled access to dialysis when needed was around 50%, but 84% of adults and 49% of children who were initiated on dialysis discontinued treatment [16]. Pooled data across the included studies revealed that at 1 year only 1% of incident (new) adult dialysis patient remained on dialysis, compared with 45% of prevalent (established, chronic) adult patients. Continuation of dialysis in much of SSA is generally dependent on the patient’s ability to continue to pay out of pocket, for dialysis, medication and transportation [16–18]. The decision to stop dialysis is usually made by the patient who simply does not return for treatment.

Physicians and nurses serving patients who require dialysis in SSA face many daily challenges regarding who needs, should receive, and can receive dialysis, how to communicate these choices, and how to respect and comfort the patient throughout [19, 20]. Nephrologists therefore have first-hand experience of how decisions to manage patients with kidney failure are made, and how resources are distributed. The experiences of these health workers have however not been well explored.

This study set out to obtain an overview of the challenges faced by nephrologists in sub-Saharan Africa in managing patients who need dialysis. Our specific focus was to investigate if and how physicians respond to bedside rationing situations.

## Methods

A survey was conducted among a randomly selected group of nephrologists from SSA. Nephrologists in SSA are few, and in order to reach them, we used the opportunity to invite participants from SSA who attended the meetings of the African Association of Nephrology (AFRAN in 2017, Yaounde, Cameroon) and the Kenyan Renal Association (KRA in 2017, Mombasa, Kenya). The questionnaire was based on a previously validated survey instrument used among physicians in Ethiopia [6]. Most questions were retained from the original survey, although examples given were changed to relate to dialysis instead of other circumstances (Supplementary File). Questions sought to investigate the frequency of specific conditions or experiences within the 2 years prior to the survey. The survey was available in English and French (the 2 official languages of the AFRAN). At the AFRAN meeting 80 paper surveys were distributed in the conference bags for attendees, 20 surveys were distributed at the KRA meeting. Survey participation was voluntary and anonymous. Formal ethics approval was waived by the Cantonal Ethics Committee of Zürich (Req-2017-00135). Survey responses were manually entered into Survey Monkey for descriptive analysis. Results are reported as proportions within each response category.

## Results

### Participant characteristics

Forty completed responses were received from nephrologists practicing in 15 sub-Saharan African countries. Eighty percent of the respondents were male, 61% were aged 36–55 years and 74% had > 10 years of medical practice experience (Table 1). Most respondents worked in government and/or teaching hospitals (62%) in addition to private practice (32%). The majority of respondents spent > 20 hours per week working in government (59%) and/or teaching hospitals (77%). Ten percent of respondents managed children under age 18 years. Forty percent of respondents participated in resource allocation decision-making in their institutions. Thirty percent of respondents reported that some form of policy existed regarding dialysis for patients with AKI or ESKD in their countries.

**Table 1:** Demographics and experience of Survey respondents

Respondent demographics/experience	Number of responses (% of responses)
**Gender**	
Male	31 (77.5)
Female	8 (20)
Not stated	1 (2.5)
**Age**	
25–35 years	7 (17.5)
35–45 years	14 (35)
46–55 years	9 (22.5)
> 55 years	8 (20)
Not stated	1 (2.5)
**Location of undergraduate training** (number of countries)	
Africa	14 (82.4)
Europe	1 (5.9)
North America (including Cuba)	1 (5.9)
Not stated	1 (5.9)
**Location of postgraduate training** (number of countries)	
Africa	9 (52.9)
Europe (including Norway)	4 (23.5)
North America, Cuba	2 (11.8)
Asia	1 (5.9)
Not stated	1 (5.9)
**Years since medical school graduation**	
< 5 years	1 (2.5)
5–10 years	9 (22.5)
11–20 years	16 (40)
> 20 years	12 (30)
Not stated	2 (5)
**Years in Nephrology**	
< 5 years	15 (37.5)
5–10 years	12 (30)
11–20 years	7 (17.5)
> 20 years	5 (12.5)
Not stated	1 (2.5)
**Current clinical role** (> 1 role possible)	
Trainee	2 (5) [nephrology fellow]
Specialist	36 (90) [2 Internists, 4 pediatric nephrologists, 30 nephrologists]
Other	3 (7.5) [teaching, research health management]
Not stated	1 (2.5)
**Location of nephrology practice** (> 1 location possible)	
Government institution	18 (45)
Teaching institution	23 (57.5)
Private for profit institution	13 (32.5)
Private section within a government facility	3 (7.5)
Private non profit institution	2 (5)
Own private institution	6 (15)
Other	1 (2.5)
Not stated	1 (2.5)
**Position in academic medicine**	
Not in academics	8 (20)
Junior faculty	12 (30)
Assistant or Associate Professor	7 (17.5)
Senior faculty/ Full Professor	8 (20)
Not stated	5 (12.5)

### Volume of patients seen with kidney disease

**Fig. 1:**
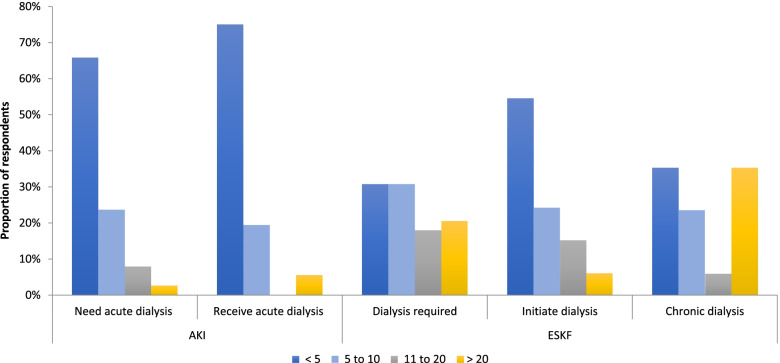
Volume of patients requiring dialysis seen by respondents per week. Proportion of respondents who reported seeing patients with acute kidney injury (AKI) and end-stage kidney failure (ESKF) per week, and the average numbers per week who could access dialysis when needed over the prior 2 years (*n* = 39 responses)

The majority of respondents (> 70%) saw at least 11 patients with kidney disease in the outpatient clinics every week (20% saw 11–20 patients, 50% saw > 20 patients). Most respondents reported seeing more patients who needed dialysis than who could receive dialysis each week: 34% of respondents saw > 5 patients who required dialysis for AKI and 69% saw > 5 patients with ESKD per week, but 75 and 54% of respondents reported that < 5 patients per week were initiated on dialysis for AKI or ESKD respectively (Fig. 1). The majority of patients seen with AKI were aged 18–60 years, whereas those seen with ESKF were aged 31–60 years (Supplementary Fig. 1).

### Access to dialysis

**Fig. 2:**
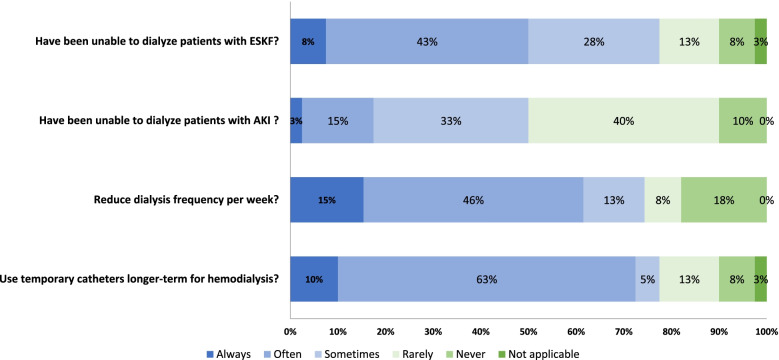
Frequency of inability to provide dialysis or need to reduce quality of dialysis. Proportion of respondents reporting the frequency with which they have been unable to dialyze patients with acute kidney injury (AKI) and end-stage kidney failure (ESKF) and the frequency with which clinical compromises were undertaken to increase access to dialysis over the prior 2 years (*n* = 40 responses)

Overall, 50 and 78% of respondents reported that within the last 2 years they had frequently (always, often, or sometimes) been unable to dialyze a patient with AKI or ESKF respectively (Fig. 2). When dialysis was provided, clinical “compromises” were often implemented, including reducing dialysis frequency per week, which reduces out of pocket costs and permits scheduling of more patients, and use of temporary catheters even for longer term dialysis, likely because of prohibitive additional costs or unavailability of more permanent dialysis access (Fig. 2). The majority of respondents reported frequent limitations in access to basic diagnostics and medications, coupled with frequent concerns regarding high out-of-pocket expenses for patients (Supplementary Fig. 2).

### Factors affecting access to dialysis or not

**Fig. 3:**
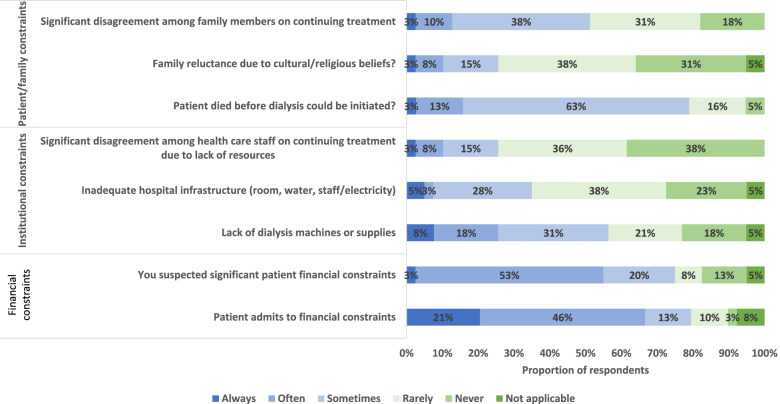
Frequency Barriers to access to dialysis encountered. Proportion of respondents reporting the frequency with which they experienced patient/family, institutional or financial constraints as barriers to provision of dialysis over the prior 2 years (*n* = 40 responses)

Patient factors which limited access to dialysis always, often or sometimes included financial constraints (reported by 79% of respondents), patients dying before dialysis could be initiated (79%), family reluctance due to cultural or religious beliefs (26%) or disagreement between family members about dialysis (51%) (Fig. 3). Additional factors which always, sometimes or often impacted a decision to initiate dialysis were the patient being a breadwinner (reported by 56% of respondents), pressure from the family to obtain dialysis (54%). Factors impacting decisions not to initiate included a patient’s poor chance of survival (reported by 78% of respondents) and living distance from the dialysis center (51%) (Supplementary Fig. 3). Half of respondents (53%) reported that pressure from families impacted dialysis decisions always, often or sometimes. Other patient factors, including patient age, social status, life-style, cognitive impairment, being the mother of small children, or likelihood of working again did not appear to systematically impact decisions to offer dialysis (data not shown).

**Fig. 4:**
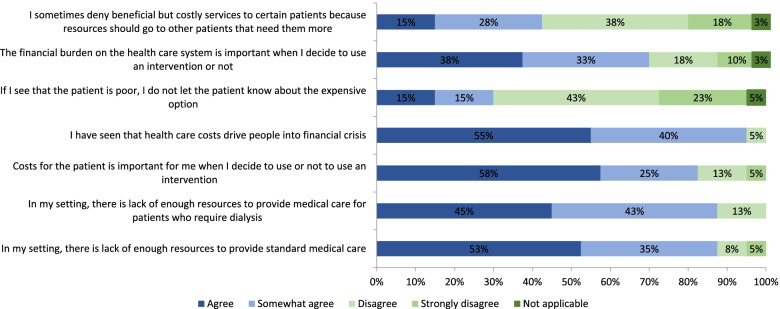
Degree of agreement or disagreement with the following statements regarding decision and resource availability. Proportion of respondents reporting the frequency with which they experience situations or circumstances which impact decision-making around provision of dialysis over the prior 2 years (*n* = 39 responses)

Institutional constraints occurring always, often or sometimes which impacted the offering of dialysis included lack of dialysis machines or supplies (reported by 56% of respondents), inadequate infrastructure or staff shortages (35%), or disagreement within the health care team regarding dialysis provision due to lack of resources (26%) (Fig. 3). Eighty-eight percent of respondents agreed or somewhat agreed that there were insufficient institutional resources to provide both standard medical care and dialysis, whereas 70% of respondents agreed or somewhat agreed that the cost to the health system was an important consideration in offering dialysis to patients (Fig. 4).

The source of payment impacted whether dialysis was offered to a patient always, often or sometimes if the patient had private insurance (reported by 50% of respondents), if there was government coverage for dialysis (60%) or if out of pocket payment for dialysis was required (78%) (Supplementary Fig. 4).

### Nephrologists’ experiences of challenges in access to dialysis

**Fig. 5:**
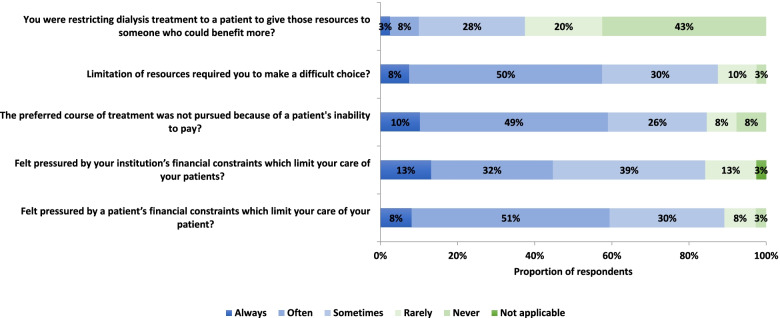
Frequency of resource scarcity limiting access to dialysis. Proportion of respondents reporting the frequency with which resource scarcity limited their ability to provide dialysis over the prior 2 years (*n* = 39 responses)

Ninety-five percent of the respondents agreed or somewhat agreed that they have seen patients plunged into financial crisis as a result of health care costs, and 36% reported frequent concern that the patients’ need for treatment was not compatible with the family’s needs or welfare (Fig. 4). Respondents reported always, often or sometimes feeling pressured by patients’ financial constraints (89%) and feeling pressured by their institution’s financial constraints (84%) (Fig. 5). Overall, the patient and institutional constraints resulted in respondents frequently (88% reported always, often or sometimes) having to make difficult choices. The majority of respondents (85%) reported frequently limiting the preferred course of treatment and at times (38% of respondents) having to make decisions to restrict dialysis for one patient in favor of another (Fig. 5). Thirty-eight percent of respondents reported often or sometimes being in doubt as to whether they should disclose the expensive treatment options for kidney failure to the patient. Given the frequent late presentation of patients with kidney failure in sub-Saharan Africa, 58% of respondents reported often or sometimes having to make decisions themselves for the patients. Some ethical dilemmas experienced by respondents are highlighted in Table 2.

**Table 2:** Examples of free text comments describing situations that may lead to moral distress

Patient constraints • “what do we do with children with AKI who have no financial means, because we could save them?” (R4) • “start dialysis for patients who are not able to continue chronic dialysis because of poverty”(R15) • “…a child had been on PD for 6 weeks with no improvement. The decision as to stop PD and palliate” (R17) • “patient’s family or relatives requesting you to do haemodialysis in terminal cases, cancer etc.” (R24) Institutional constraints • “no resources available” (R1) • “I had to stop dialysis despite no recovery because we have no place in chronic dialysis”(R13) • “there is no public dialysis in my country. Diagnosis is made very late. I struggle to fight for prevention”(R8) • “the dialysis budget is badly used, Corruption ++”(R7) • “Often politicians will interfere with our guidelines on provision of dialysis” (R29) Physician constraints/strategies • “when a patient is being managed in another health facility comes to me I find it difficult to decide where my loyalty lies. To the patient to divulge all the info or to the doctor and I hide things under the carpet?” (R2) • “the patient has dementia, family finds resources for dialysis with difficulty. What do I do?”(R13) • “Our own renal unit have established committee to decide which patients would be offered the RRT. We have entry and even exit criteria for our haemodialysis programme”(R27) • “We have regular meetings with decision makers and stakeholders”(R29) • “It is a huge challenge to work as a nephrologist in Africa but with international support from organisations like AFRAN etc. lobbying for a lot of services to be implemented in possible” (R13) • “I think transplantation is the good therapy to take care end stage of CKD in our countries - then promote that therapy. Develop a program of screen and prevent CKD which can be proposed in Africa” (R11)a	

Figure 5 shows nephrologists’ reactions to the ethical dilemmas they face. Seventy-four percent of respondents always, often or sometimes felt burdened by ethical dilemmas and worried about patients out of hospital hours. As a consequence of resource scarcity, almost 46% reported frequently regretting their choice of profession and 26% had considered leaving the country. Seventy-four percent of respondents did however report having frequent discussions with colleagues about ethical dilemmas.

## Discussion

Physicians managing patients who require dialysis in SSA frequently face resource scarcity and report that patients don’t get the treatment they need. Both patient and institutional constraints impact whether nephrologists may offer dialysis to a patient, and how dialysis may be delivered. Ethical dilemmas therefore frequently arise, in terms of ability to provide dialysis, weighing benefits versus harm for the patient in terms of clinical care, weighing benefits versus harm for their families in terms of psychosocial and economic well-being, and weighing benefits versus harm between patients with similar needs. This survey also highlights the tension in navigating between the patient’s resource limitations and those of the institution. The African nephrologists surveyed reported playing conflicting roles, where they cannot focus entirely on the patient at hand, but must consider simultaneous needs of other (sicker? or more salvageable?) patients, and the broader health system resources, in situations where institutional/government guidelines for resource allocation at the bedside are lacking. As a result, the majority of the nephrologists feel burdened by ethical dilemmas, and almost half of them regret their choice of profession due to this.

### Nephrology in resource limited contexts

This study reflected what has been reported by others in SSA. Patients with kidney failure are younger and often have fewer co-morbidities compared with those in less resource deprived settings, and diagnosis tends to be made late due to suboptimal access to quality health care [21]. Even if risk factors for kidney disease or kidney disease itself are diagnosed early in SSA, access to appropriate and long-term essential diagnostics and treatment is often limited [9]. Because kidney disease requires awareness among the (overworked and under-resourced) health care workers, and access to reliable laboratory testing for diagnosis, the burden has been underappreciated in many settings and kidney disease has therefore not been seen as a priority [22].

Kidney failure, which requires dialysis or transplantation as treatment, is notoriously unaffordable for patients and governments alike. This may have contributed to its being relatively overlooked in essential health care packages in many settings [10, 14]. Given the high costs of dialysis, many multiples of the gross domestic product per capita and therefore not considered “cost -effective” [23, 24], universal access to dialysis is not included in most low-resource country health care packages [14, 25, 26]. The consequence of this policy is seen among our respondents who report that both diagnostics and therapeutic options are limited for the patients and that the government doesn’t provide sufficiently for patients with kidney disease. Knowing that nephrologists are scarce in SSA, the number of patients with kidney disease seen by nephrologists likely reflects the tip of the iceberg [9, 14, 23–38].

### Bedside priority setting

The major reasons why patients do not receive optimal dialysis treatment or discontinue the treatment appear to be a lack of resources and infrastructure in the hospital itself and the patients’ inability to pay. Almost all study participants reported having to make decisions to limit services to save resources for the patient or their institution, suggesting that clinicians are weighing not only the clinical and financial well-being of the patent, but are having to be mindful of the broader resources. Reduction of dialysis frequency is a reality for many in resource limited settings [27, 28]. Such an approach of “sharing” health care resources highlights the dilemma between achieving quality and increasing equity which is a daily struggle as highlighted by the respondents in this study. These compromises, considered a double standard, are generally rejected by bodies such as the World Health Organization, but may be considered ethical under certain circumstances if the compromise remains effective [29, 30].

### Choosing among patients

More than 1/3 of our participants reported having to choose among patients for access to dialysis, when resources were not sufficient to treat all in need. This priority dilemma has been much discussed during the Covid-19 pandemic and leads to discussions about which criteria should be given more weight in these decisions [39]. Survey respondents reported giving weight to various medical and non-medical factors in making decisions about dialysis allocation. The potential benefit of treatment was important to 78% of the participants, where decision-making depended on a patient’s poor chance of survival. Priority setting based on how effective a treatment is for a condition, measured in healthy life years is well-accepted [40]. Indeed, during the COVID-19 pandemic, the topic of “futility” in medicine has been much discussed, and most triage guidelines for access to ICU beds suggest prioritization of patients with a better prognosis over other criteria [3]. What makes the decision-making much more harsh and ethically problematic for nephrologists in SSA, is that many patients are young and some have AKI which could be reversible with a few sessions of dialysis, therefore the concept of “futility” is less clear than in the face of a deadly pandemic. Although not directly asked in this survey, especially with AKI, when treatment is available but the patient cannot afford it, physicians have sometimes paid for dialysis themselves (personal communication GA). This is different for ESKF, however, where conservative care has now been accepted as an alternative when there is no access to dialysis. Initiating dialysis in a patient only to stop after a few sessions because of inability to pay or eat is catastrophic for all involved.

### Steward of family economy (financial risk protection)

Our study confirms that when not provided by the government, patients and families, even if they cannot afford it, try to pay for the service themselves. The reality of how this leads to catastrophic health expenditures is well known to the survey respondents, with 30% reporting that at times they may try to protect patients or families from these expenses by not informing them about the treatment options or not offering them the opportunity to start dialysis treatment. When access to dialysis is reliant on out-of-pocket expenditure, patients either forgo or stop therapy early on, or experience high rates of catastrophic health expenditure [16, 17, 41–43]. Such an approach strongly disfavors the poor, as well as reduces access for most of the population due to the prohibitive costs. Clinicians attempt to reduce costs by reducing dialysis frequency (which may be safe, at least over the short term, in selected patients [44]) or relying on temporary catheters for dialysis (which are cheaper but carry higher risk of complications [45]). These clinical compromises, although suboptimal, may be the only way to keep an individual alive.

In addition to the patient’s ability to pay or not, other criteria such as the patient being a breadwinner and geography were also taken into consideration by over half of the respondents in making dialysis allocation decisions. Giving weight to such personal factors as breadwinner status are by some viewed as unfair, as this tends to favor men with income [46]. However, in settings such as SSA, not giving priority to the family member with income will devastate whole families, and should be avoided if other concerns, like financial risk protection, are prioritized. Families themselves often make this decision before seeking care, which may contribute to the greater proportion of men being treated for ESKF in SSA [47–49].

Consideration of criteria such as geographic distance from a dialysis center may be pragmatic, but is contested as this will exacerbate inequities [46, 50]. Peritoneal dialysis would get around this barrier, however this form of dialysis is not often available for chronic dialysis in most of SSA for many reasons, and is unaffordable out-of-pocket for most patients [51, 52].

While we may blame the health system or states in some cases, the hospital/health center is not the first stop for many patients. Many patients will go for alternative therapy, and some will fall into the hands of incompetent healthcare-providers. States must therefore also develop education programs for the general public and healthcare providers regarding kidney health, even in the absence of dialysis, as these are most cost-efficient.

### Moral distress

Since being first formulated by Andrew Jameton, a nurse-philosopher in 1984, the concept “moral distress” has been debated [53–55] and a broader definition of moral distress has emerged to include “a specific psychological response to morally challenging situations such as those of moral constraint or moral conflict, or both” [53]. The initial concept of moral distress grew out of nursing, where nurses in high income settings felt pressure to continue care in patients where they felt there was little benefit and prolonging of suffering. The moral discomfort arose from provision of *too much* care. In high income settings such circumstances are often referred to as *futile care*. In sub-Saharan Africa and many other resource limited settings in contrast, moral distress arises out of the converse problem – there is *too little* care available for people who would benefit considerably. The ethical dilemmas and moral constraints reported by nephrologists in SSA in this survey are clear and are largely based on the lack of financial and physical resources of both patients and institutions, which preclude optimal treatment of many patients. These challenges therefore fit the broader definition of moral distress.

Factors associated with moral distress in clinicians, include a poor ethical climate, lack of interdisciplinary collaboration and peer support, work load (high and low patient loads), working in acute care, feeling of disempowerment, low levels of health care worker autonomy, and lack of opportunities for ethical debate [56]. Pressure from families is an additional factor reported by nephrologists in this survey. Our respondents therefore experience several moral conflicts, and struggle to balance conflicting ethical principles of autonomy, benefit, reducing harm in individual cases and justice to the broader community. Critical decisions must be taken, often acutely, on a background of chronic health system resource limitations, which in many cases have contributed to the late diagnosis through poor access to prevention or early treatment of kidney disease.

The concept of moral distress has not been well studied in nephrology and even less in SSA [57]. A study among nurses who manage patients with chronic kidney disease in 2 major hospitals in Nigeria found that the burden of care, as measured by the Zarit Burden of Life Instrument, was highest for dialysis [58]. Leading factors which contributed to the higher burden of care in dialysis included staff shortages, patients’ lack of funds and difficulty finding kidney donors, closely followed by erratic power and water supplies, lack of equipment, patient mal-adherence and patients’ inability to sustain dialysis. Dialysis in Nigeria is largely paid for out of pocket which is not sustainable over the long term for most patients. A study from Guatemala, where dialysis is government funded, but geographically and infrastructurally limited, also reported a high degree of moral distress among clinicians providing dialysis [27]. In this setting the demand for dialysis is higher than the supply, resulting in clinicians needing to triage patients daily. On average dialysis was delivered every 7–10 days, and patients had to demonstrate an indication for dialysis on a given day [27]. Clinicians viewed themselves as “a little soldier in the ranks of combat”. Interviewees described feelings of guilt, shame, sadness, disempowerment, exhaustion and devaluation. Among clinicians who remained working in the system, however, resilience was buoyed by being able to help some patients [27].

### The burden of being a nephrologist

**Fig. 6:**
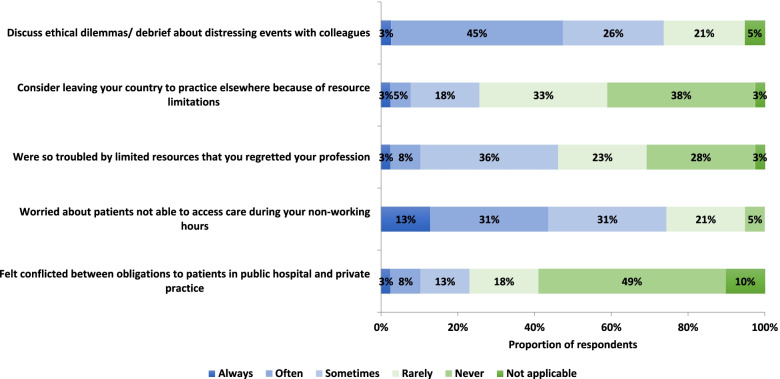
Frequency of moral dilemmas. Proportion of respondents reporting the frequency with which they experienced and were able to share moral dilemmas relating to the provision of dialysis over the prior 2 years (*n* = 39 responses)

Moral distress is associated with reduced job satisfaction and engagement, higher risk of burnout, poorer patient outcomes and reduced patient satisfaction [59, 60]. In the current study, 3 in 4 nephrologists worried about patients during non-working hours, one in 2 sometimes regretted their choice of profession and one in 4 had considered leaving their country over in the past 2 years due to the resource limited setting (Fig. 6). Being forced to provide second best or no treatment at all for patients with conditions that could easily and cheaply have been prevented, is stressful and can be a driving force for wanting to leave the profession and even country (brain-drain) [6]. There are no similar studies of nephrologists elsewhere that we are aware of, but the proportion of respondents regretting their choice of profession seems high compared to studies among physicians from the high-income settings. In a study among US physicians over 70% would choose their specialty again, while another study showed that only 12,2% of the internal medicine residents regretted their choice of profession (14.1% of the total residents regretted) [61, 62]. .When compared to the level described in a study among general physicians in Ethiopia, however, where 74% regretted their choice of profession at least weekly, the level among nephrologists appears lower [6]. The resource -scarcity and the frequent need to make difficult choices reported by the survey participants, are similar to those reported in the Ethiopian study. This difference could have multiple interpretations. Once patients reach a nephrologist, they have already passed the hurdles of access to care and diagnosis. It is possible therefore that specialists such as nephrologists may be relatively shielded from some of the every-day resource constraints experienced by generalists. In addition, more senior physicians, as many in the current study were, may be more able to mobilize resources, especially if they also have private practices. On the other hand, it is also possible (although we presume very unlikely) that more senior physicians may have become accustomed to the resource limitations and develop a more nihilistic attitude and are less disturbed by the resource limitations. Experienced nephrologists may however be choosing to remain in their countries and institutions, as their advocacy efforts over time may be beginning to bear fruit, and they may feel a growing sense of purpose, given that dialysis services are expanding, and governments are beginning to consider how to address the challenges. Greater numbers of nephrologists are being trained locally and are adapted to these challenges. Their choice may be a way to bring solutions.

### Guidelines and needs

There are some well-described factors which may prevent moral distress, including having a good ethical climate at work and the ability to reflect with colleagues. Importantly, in this survey, despite the high degree of moral distress reported, 3 in 4 nephrologists did report frequently engaging with colleagues to discuss and work through the dilemmas. The burden of having to ration care on a case-by-case basis currently placed on individual shoulders may be reduced by development/implementation of national/institutional guidance on resource allocation [31]. Most respondents in this study articulated that guidance on dialysis allocation was lacking, and that guidelines are needed to improve transparency of criteria governing access to dialysis, and consistency and fairness of decision-making at the bedside, when resources are limited. Transparent priority setting at the national level is also required [22]. Such guidance is however largely lacking in SSA. In the Western Cape in South Africa, in response to tangible inequities in access to state-funded dialysis (restricted to patients who are eligible for transplantation), criteria for dialysis eligibility have been developed using the Accountability for Reasonableness framework, which includes multi-stakeholder and community engagement and emphasizes transparency [63]. The fairness of these guidelines is however debated elsewhere in South Africa, given that socioeconomic factors still heavily impact decision-making, and that high inequity remains between the public and the private sectors [37]. When budgets are constrained, however, some criteria must be in place to support some level of justice in resource allocation. Withholding a therapy where no clinical benefit is expected is largely acceptable [40], but even objective clinical indications must be transparently communicated to all stakeholders. When resource allocation decisions reach the realm of rationing (i.e. withholding a therapy from one individual or group in order to benefit another), transparency, explicitness and public accountability become critically important, both for the patient and for the bedside clinician [40]. Such decisions based on unaffordability may be justifiable and acceptable, but they should be communicated to patients as such, and not “disguised as “clinical” decisions” [46, 64, 65].

Dialysis is becoming more available in many countries in SSA, and in some countries, it is subsidized by the state. Many more patients are now receiving dialysis. The increase in dialysis facilities is directly related to the increase in number of nephrologists. The increase in nephrologists in SSA has been largely due to funding and development of training centers supported by the International Society of Nephrology. The increasing number of dialysis centers, has taken dialysis nearer to the people, thus reducing out of pocket costs, like transportation. Without nephrologists, kidney care and dialysis will not grow, therefore attention must be paid to the moral dilemmas and challenges presented here to support and retain nephrologists in SSA.

### Strengths and limitations

The current study has several limitations. The sample size was small and included nephrologists from only 15 countries in sub-Saharan Africa. Also, most patients with kidney disease do not get to see a nephrologist, as nephrologists are few in SSA, and most patients are treated by non-specialists. The results presented here therefore reflect dilemmas associated with the care of those who reach the specialists. The findings can therefore only provide a glimpse into the real-world challenges faced by patients and providers in settings where dialysis may exist, but is not accessible to all. The findings require further validation and study in larger samples stratified by diverse institutional environments. The response rate of 40% is also a potential limitation, especially since the participants came from diverse settings across the continent. Given the relative consistency of responses between respondents however, it is likely that these are representative of common ethical dilemmas experienced in nephrology practice in sub-Saharan Africa. Surveys were distributed in conference bags, together with several other leaflets, therefore it is possible that some conference attendees did not recognize the survey for what it was. A major strength of our study is that it is the first one of its kind in exploring ethical dilemmas experienced among nephrologists in SSA. Inclusion of nurses and other providers as well as patients and their families in future studies will give a more comprehensive picture of the myriads of ethical challenges in renal care. The impact on mental health and wellbeing of nephrologists and allied clinicians in SSA was not specifically addressed in this study. Indirect evidence gathered here, that nephrologists felt pressured by the decisions they had to make and worried about patients outside of hospital hours (Figs. 5 and 6) suggests there may be an important impact. This should be addressed in future studies.

## Conclusion

Nephrologists in SSA manage patients who require dialysis under severe resource constraints, and patient and institutional constraints impact whether, if, and how dialysis may be delivered. They report how patients are lost due to inability to pay, and how families are burdened /succumb to catastrophic health expenditures in trying to pay for dialysis. Conflicting interests and ethical dilemmas must be handled, and the nephrologist must prioritize and weigh benefits versus harm for and between patients in terms of clinical care, for their families, and for the health care institutions. As a result, most nephrologists feel burdened by ethical dilemmas, and almost half of them regret their choice of profession due to this. Improving public health prevention strategies and sustainable and equitable access to primary care for early diagnosis and life-long quality treatment and follow-up are the obvious low-hanging fruit to reduce the future burden of kidney disease. How to handle that millions are dying now because they cannot access dialysis must however also get more attention. The COVID-19 pandemic is forcing the world to reckon with major global and national inequities in disease risk and access to care. Such inequities pre-date and are much broader than COVID-19. The clear dependence on a patient’s resources and infrastructural inadequacies in determining access to dialysis in sub-Saharan Africa is an important concern that must be addressed if moral distress is to be reduced among dialysis care staff, and if equity of access to dialysis is to improve.

## Supplementary Information

**Additional file 1.**

**Additional file 2: Supplementary Fig. 1.** Average age group (years) of patients seen by survey respondents. Proportion of respondents reporting the frequency with which they saw patients with AKI or ESKD and their age distributions over the prior 2 years (*n* = 39 responses). **Supplementary Fig. 2.** Concerns regarding access to medication and laboratory testing. Proportion of respondents reporting the frequency with which they experienced limitations in access to medication or laboratory testing due to cost or availability over the prior 2 years(*n* = 39 responses). **Supplementary Fig. 3.** Factors influencing decision-making regarding dailysis allocation. Proportion of respondents reporting the frequency with which various patient-related factors impact the decision to provide, or access to, dialysis over the prior 2 years(*n* = 39 responses). **Supplementary Fig. 4.** Sources of payment for dialysis. Proportion of respondents reporting the frequency of types of financial coverage for dialysis over the prior 2 years(*n* = 39 responses).

## Data Availability

All data generated or analysed during this study are included in this published article and its supplementary information files. Survey is available in the Supplementary data.

## Abbreviations

SSA: Sub-Saharan Africa
NCD: Non-communicable disease
AKI: Acute kidney injury
ESKF: End-stage kidney failure
