# Supplementary material for: Bedside rationing and moral distress in nephrologists in sub- Saharan Africa
Source: BMC Nephrol. 2022 May 25;23:196. doi: 10.1186/s12882-022-02827-2 (PMC9131991; doi:10.1186/s12882-022-02827-2)
Supplement: Supplementary file 1 — Additional file 1. [file 12882_2022_2827_MOESM1_ESM.pdf]

Challenges in Nephrology: A Survey of Physicians Regarding Ethical Dilemmas in Clinical Practice

This survey is anonymous. Thank you for your participation! Your contribution is highly valued, as getting information from physicians working "on the ground" with patients is crucial in this first investigation of medical ethical dilemmas in nephrology in resource limited settings. The data from this survey will also be useful to inform evidence-based policy and practice in medical ethics and may be used for teaching of medical students and for academic publication to disseminate the findings. Since there are important differences between health care levels, some questions may not seem applicable to you, but please answer as best you can based upon your situation. We value your opinions, and are grateful for your participation. We will inform you of the outcomes of the survey after completion by general email.

If you have any questions or comments or which to participate further in this project, please contact us at vluyckx@hotmail.com (Valerie Luyckx) or maglo09@hotmail.com (Gloria Ashuntantang)

By filling in and returning this survey we presume you consent to our inclusion of your anonymous responses in the study.

Part 1. Background

1. What is your gender?

- ◯ Female
- ◯ Male

2. What is your age?

- ◯ < 25 years
- ◯ 25 to 35 years
- ◯ 36 to 45 years
- ◯ 46 to 55 years
- ◯ older than 55 years

3. In which country did you complete your undergraduate medical training?

[ ]

4. In which country did you complete your post-graduate medical training?

[ ]

5. Since graduating as a doctor how many years have you been practicing medicine?

- ◯ < 5 years
- ◯ 5-10 years
- ◯ 11 - 20 years
- ◯ > 20 years

6. How long have you been working in nephrology?

- ◯ < 5 years
- ◯ 5-10 years
- ◯ 11-20 years
- ◯ > 20 years

7. What is your current clinical role?

General practice (yes/no) [ ]

Trainee (please specify) [ ]

Specialist (please specify) [ ]

Other (please specify) [ ]

8. Where do you practice as a doctor seeing patients with kidney disease? (please indicate all that apply)

- [ ] Government institution
- [ ] Teaching institution
- [ ] Private for profit institution
- [ ] Private section within a government facility
- [ ] Private non profit institution
- [ ] Own private institution
- [ ] Other

9. If you are involved in academic medicine, what is your position?

- ( ) Not in academics
- ( ) Junior faculty
- ( ) Assistant or Associate Professor
- ( ) Senior faculty/ Full Professor

10. Do you participate in any decisions on where the resources are spent/allocated like in planning and budgeting in your health care department/facility?

- ( ) Yes
- ( ) No

Part 2: Clinical practice

11. On average, approximately how many hours/ week do you work in:

	< 5 hours	5-10 hours	11-20 hours	> 20 hours
Government institution	○	○	○	○
Teaching hospital	○	○	○	○
Private practice	○	○	○	○
Other	○	○	○	○

Other (please specify)

12. On average approximately how many patients with kidney disease do you see in a week?

	< 5	5-10	11-20	> 20
Inpatient	○	○	○	○
Outpatient	○	○	○	○

13. On average approximately how many patients per week do you see who require dialysis for:

	< 5	5-10	11-20	> 20
Acute kidney injury (AKI)	○	○	○	○
End-stage kidney disease (ESKD)	○	○	○	○

14. On average how many patients per week do you see who receive dialysis for:

	< 5	5-10	11-20	> 20
Acute dialysis for AKI	○	○	○	○
Acute dialysis for new ESKD	○	○	○	○
Chronic ESKD	○	○	○	○

15. In which average age group (years) are most of the kidney patients that you take care of?

	< 18 years	18 - 30 years	31 - 45 years	46-60 years	> 60 years
AKI	☐	☐	☐	☐	☐
New ESKD	☐	☐	☐	☐	☐
Chronic ESKD	☐	☐	☐	☐	☐

Part 3: Ethical Dilemmas

Below is a list of situations where medical decision-making can be difficult.

16. In the last two years how often have you been in the following situations in your routine clinical practice caring for patients with kidney disease?

	Always	Often	Sometimes	Rarely	Never	Not applicable
Unable to obtain basic laboratory testing you needed to appropriately diagnose and manage patients with kidney disease?	○	○	○	○	○	○
Concerned that laboratory testing is unaffordable to your patient?	○	○	○	○	○	○
Unable to obtain consistent basic medication you required for control of blood pressure and/or diabetes for patients with kidney disease?	○	○	○	○	○	○
Concerned that basic medication is unaffordable to your patient?	○	○	○	○	○	○
Felt pressured by a patient's financial constraints which limit your care of your patient?	○	○	○	○	○	○
Felt pressured by your institution's financial constraints which limit your care of your patients?	○	○	○	○	○	○
The preferred course of treatment was not pursued because of a patient's inability to pay?	○	○	○	○	○	○
You were in doubt if a diagnosis should be disclosed to the patient?	○	○	○	○	○	○

17. In the last two years how often have you been in the following situations regarding patient access to dialysis

	Always	Often	Sometimes	Rarely	Never	Not applicable
Limitation of resources required you to make a difficult choice?	○	○	○	○	○	○
Have been unable to dialyze patients with AKI who require dialysis?	○	○	○	○	○	○
Have been unable to dialyze patients with ESKD?	○	○	○	○	○	○

18. In the last 2 years, if you have been unable to dialyze a patient who required it, how often did the following barriers contribute?

	Always	Often	Sometimes	Rarely	Never	Not applicable
Patient admits to financial constraints?	○	○	○	○	○	○
You suspected significant patient financial constraints?	○	○	○	○	○	○
Lack of dialysis machines or supplies?	○	○	○	○	○	○
Inadequate hospital infrastructure (room, water, staff/electricity)?	○	○	○	○	○	○
Reluctance from staff?	○	○	○	○	○	○
Patient died before dialysis could be initiated?	○	○	○	○	○	○
Family reluctance due to cultural/religious beliefs?	○	○	○	○	○	○

19. How often do you or your patients use the following to contain costs?

	Always	Often	Sometimes	Rarely	Never	Not applicable
Reduce dialysis frequency per week?	○	○	○	○	○	○
Use temporary catheters longer-term for hemodialysis?	○	○	○	○	○	○

20. In the last two years how often have you been in the following situations?

	Always	Often	Sometimes	Rarely	Never	Not applicable
You cared for adult patients that were not in a state to make a decision for themselves (unconscious/ disabled), and you had to decide for them?	○	○	○	○	○	○
You were restricting dialysis treatment to a patient to give those resources to someone who could benefit more?	○	○	○	○	○	○
You felt that the patients need of treatment was not in agreement with the patient’s family needs or welfare?	○	○	○	○	○	○
There was significant disagreement among health care personnel on continuing treatment of the patient due to lack of resources?	○	○	○	○	○	○
There was significant disagreement among family members on continuing treatment of the patient?	○	○	○	○	○	○
You felt conflicted between your obligations to the patients in the public hospital and to your patients in private practice?	○	○	○	○	○	○
You discussed cases which presented ethical dilemmas/ debrief about distressing events with colleagues?	○	○	○	○	○	○
You worried about patients not able to access care during your non-working hours?	○	○	○	○	○	○

	Always	Often	Sometimes	Rarely	Never	Not applicable
You were so troubled by limited resources that you regretted your profession?	◯	◯	◯	◯	◯	◯
Resource limitations made/make you consider leaving your country to practice elsewhere?	◯	◯	◯	◯	◯	◯

21. To what extent do you agree or disagree with the following statements

	Agree	Somewhat agree	Disagree	Strongly disagree	Not applicable
In my setting, there is lack of enough resources to provide standard medical care	◯	◯	◯	◯	◯
In my setting, there is lack of enough resources to provide medical care for patients who require dialysis	◯	◯	◯	◯	◯
Costs for the patient is important for me when I decide to use or not to use an intervention	◯	◯	◯	◯	◯
I have seen that health care costs drive people into financial crisis	◯	◯	◯	◯	◯
If I see that the patient is poor, I do not let the patient know about the expensive option	◯	◯	◯	◯	◯
The financial burden on the health care system is important when I decide to use an intervention or not	◯	◯	◯	◯	◯
I sometimes deny beneficial but costly services to certain patients because resources should go to other patients that need them more	◯	◯	◯	◯	◯

22. How often do the following factors influence whether you offer dialysis to a patient?

	Always	Often	Sometimes	Rarely	Never	Not applicable
The patient has the possibility of receiving a kidney transplant	◯	◯	◯	◯	◯	◯
The patient has private health insurance	◯	◯	◯	◯	◯	◯
The costs of dialysis is paid by the government	◯	◯	◯	◯	◯	◯
The costs of dialysis is paid by the patient/family out of pocket	◯	◯	◯	◯	◯	◯
The patient is old > 70y	◯	◯	◯	◯	◯	◯
The patient is a neonate	◯	◯	◯	◯	◯	◯
The patient is a child	◯	◯	◯	◯	◯	◯
The patient is an adolescent	◯	◯	◯	◯	◯	◯
The patient is poor	◯	◯	◯	◯	◯	◯
The patient is an important person in society	◯	◯	◯	◯	◯	◯
The patient is the family bread-winner	◯	◯	◯	◯	◯	◯
The patient is a mother of small children	◯	◯	◯	◯	◯	◯
The patient is cognitively impaired	◯	◯	◯	◯	◯	◯
The patient lives far away	◯	◯	◯	◯	◯	◯
The patient has a poor chance of survival	◯	◯	◯	◯	◯	◯
The patient/family pushes strongly for dialysis	◯	◯	◯	◯	◯	◯
The patient will not be able to work again	◯	◯	◯	◯	◯	◯

	Always	Often	Sometimes	Rarely	Never	Not applicable
The kidney failure is a result of the patient’s life-style choices (obesity, HIV, drug abuse, herbal remedies)	◯	◯	◯	◯	◯	◯

23. Does your country have a national policy regarding dialysis?

	Yes	No	Unsure
My country has a policy governing who can receive dialysis for AKI	◯	◯	◯
My country has a policy governing who can receive dialysis for ESKD	◯	◯	◯

24. Please describe a dilemma you have encountered in your own words (without any identifiable data)? Please continue on the back of the page if needed.

25. Does the situation (-s) you have described happen often where you work?

◯ Yes, quite common

◯ Happens sometimes

◯ Happens rarely

26. Is there any support structure available in your practice to discuss ethical dilemmas? (e.g. ethics consultation, ethics committees?). If yes, please describe.

27. Do you have any further comments you wish to share?

Thank you for your participation in this survey.
