# Supplementary material for: Bedside rationing and moral distress in nephrologists in sub- Saharan Africa
Source: BMC Nephrol. 2022 May 25;23:196. doi: 10.1186/s12882-022-02827-2 (PMC9131991; doi:10.1186/s12882-022-02827-2)

Supplementary Figure 1: Average age group (years) of patients seen by survey respondents. Proportion of respondents reporting the frequency with which they saw patients with AKI or ESKD and their age distributions over the prior 2 years (n= 39 responses)

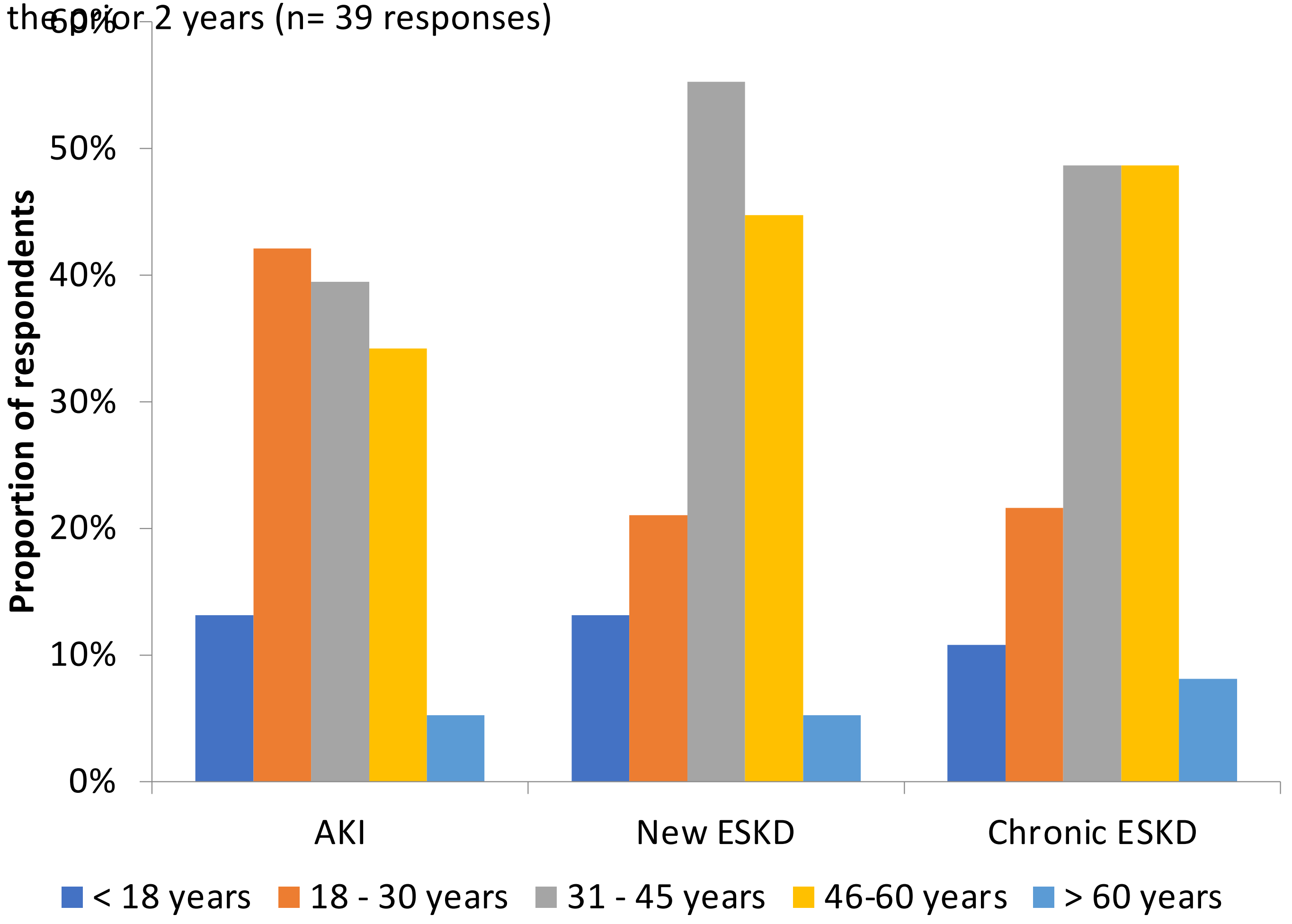

Supplementary Figure 2: Concerns regarding access to medication and laboratory testing. Proportion of respondents reporting the frequency with which they experienced limitations in access to medication or laboratory testing due to cost or availability over the prior 2 years(n= 39 responses).

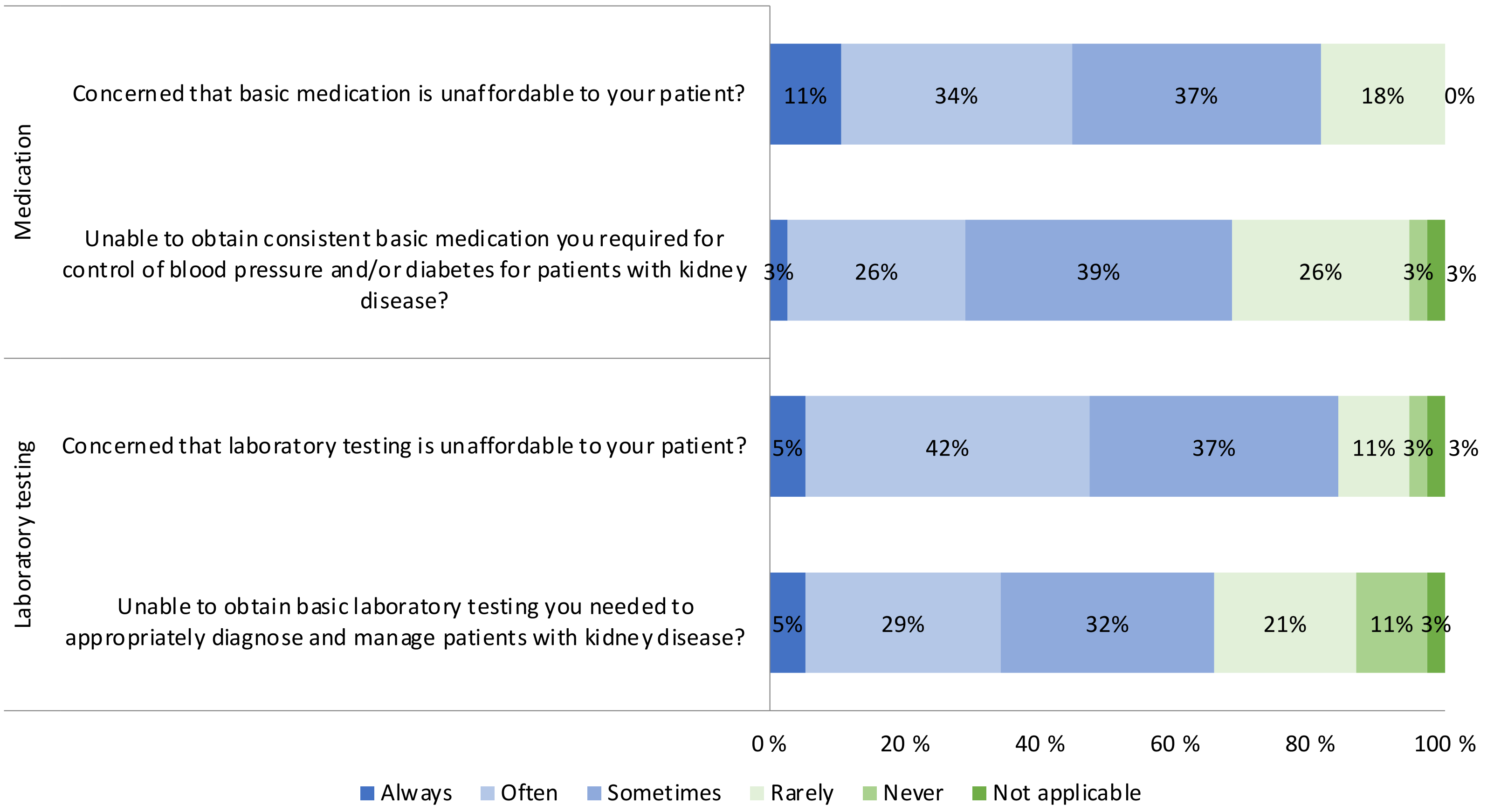

Supplementary Figure 3: Factors influencing decision-making regarding dailysis allocation. Proportion of respondents reporting the frequency with which various patient-related factors impact the decision to provide, or access to, dialysis over the prior 2 years(n= 39 responses).

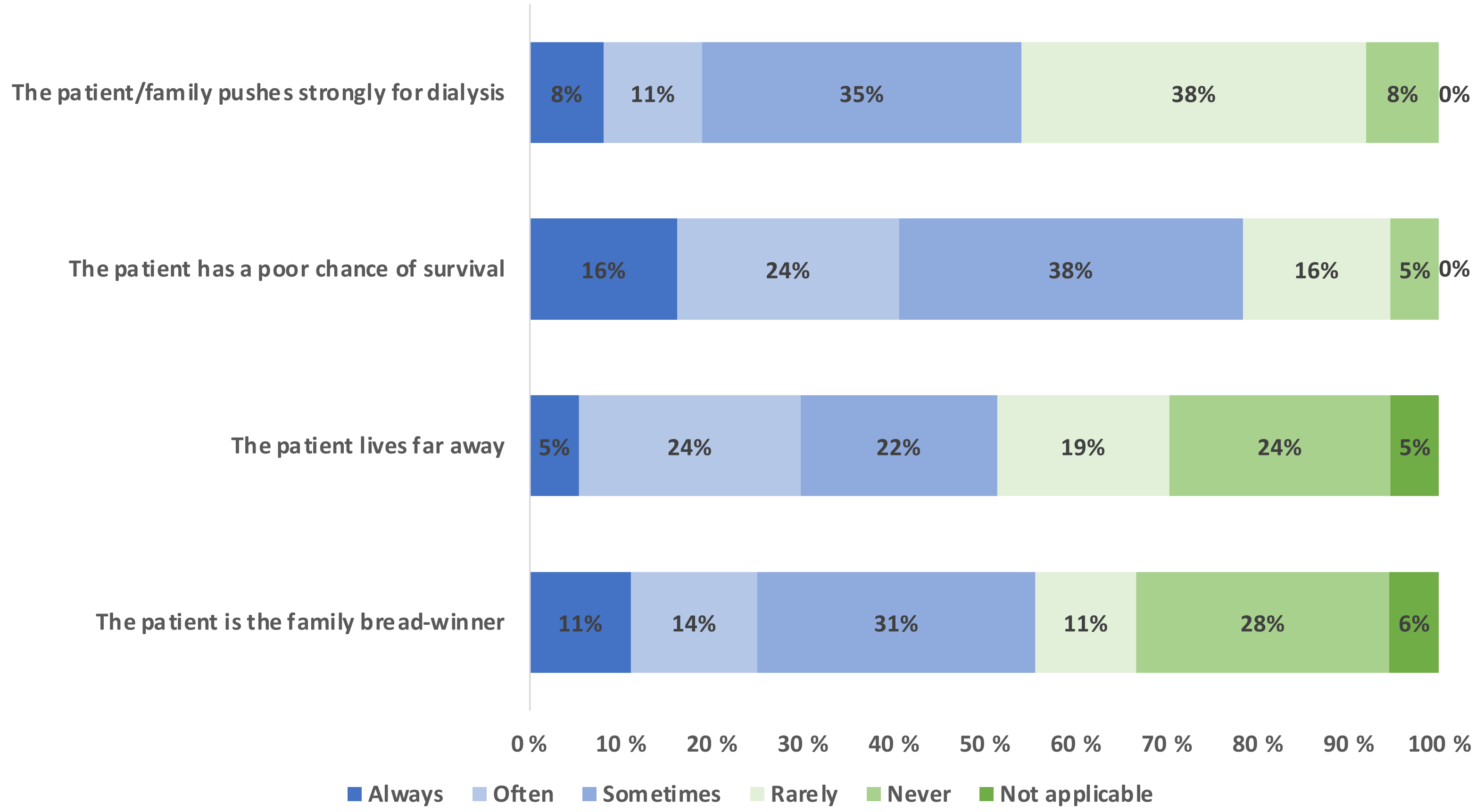

Supplementary Figure 4: Sources of payment for dialysis. Proportion of respondents reporting the frequency of types of financial coverage for dialysis over the prior 2 years(n= 39 responses).

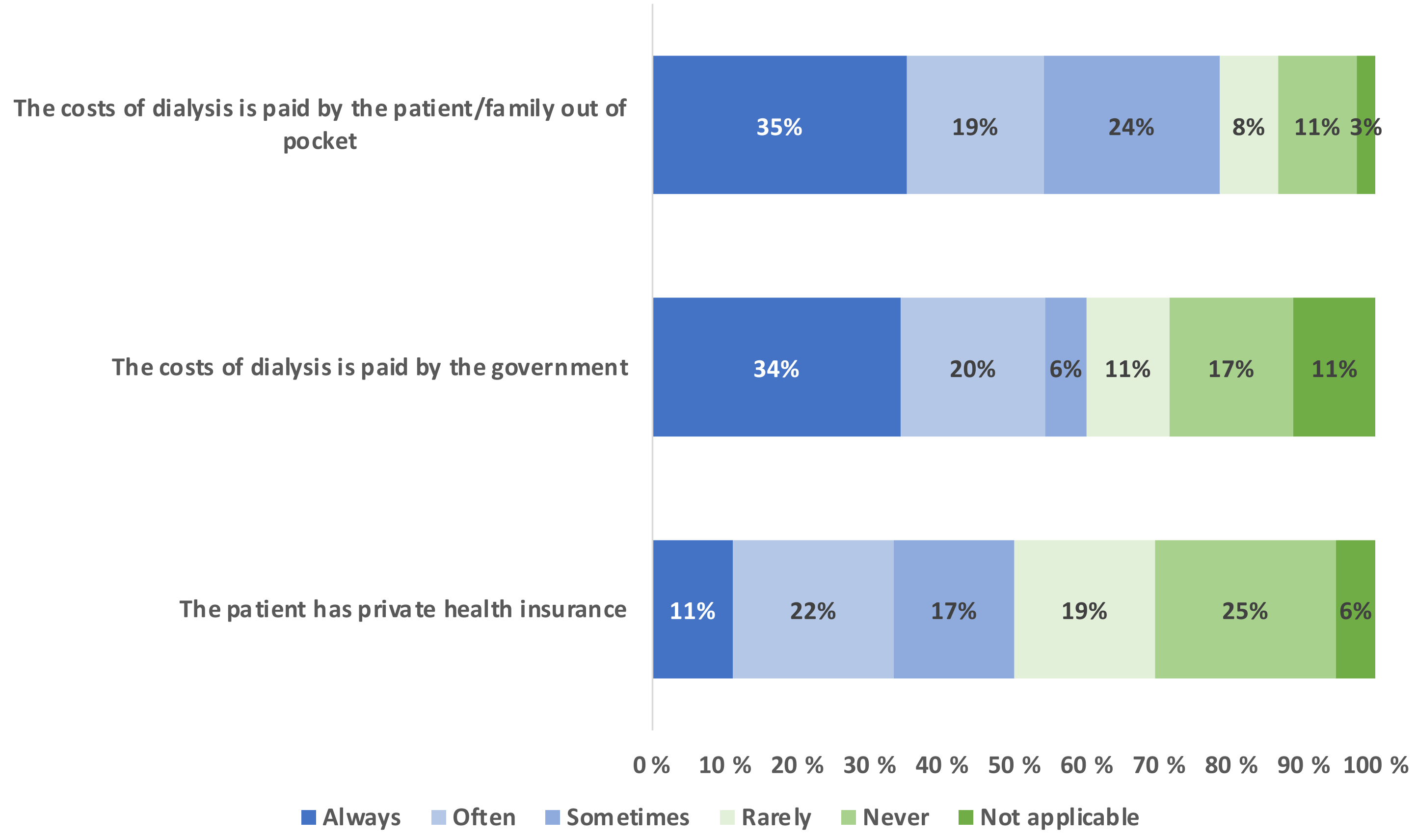

Supplement: Supplementary file 2 — Additional file 2: Supplementary Fig. 1. Average age group (years) of patients seen by survey respondents. Proportion of respondents reporting the frequency with which they saw patients with AKI or ESKD and their age distributions over the prior 2 years (n = 39 responses). Supplementary Fig. 2. Concerns regarding access to medication and laboratory testing. Proportion of respondents reporting the frequency with which they experienced limitations in access to medication or laboratory testing due to cost or availability over the prior 2 years(n = 39 responses). Supplementary Fig. 3. Factors influencing decision-making regarding dailysis allocation. Proportion of respondents reporting the frequency with which various patient-related factors impact the decision to provide, or access to, dialysis over the prior 2 years(n = 39 responses). Supplementary Fig. 4. Sources of payment for dialysis. Proportion of respondents reporting the frequency of types of financial coverage for dialysis over the prior 2 years(n = 39 responses). [file 12882_2022_2827_MOESM2_ESM.pdf]
